# Norepinephrine with dopamine infusion on the end-tidal carbon dioxide (ETco2) pressure in patients with septic shock

**DOI:** 10.22088/cjim.12.4.580

**Published:** 2021

**Authors:** Mohammad Sazgar, Iraj Golikhatir, Seyedeh Masoomeh Pashaee, Fatemeh Tirandaz, Abolfazl Firouzian, Hamed miniahidashti

**Affiliations:** 1 Department of Emergency Medicine, Mazandaran University of Medical Sciences, Sari, Iran; 2 Department of Anesthesiology, Division of Intensive Care Unit, Mazandaran University of Medical Sciences, Sari, Iran

**Keywords:** Septic shock, End tidal carbon dioxide, Norepinephrine, Dopamine

## Abstract

**Background:**

Septic shock is a critical medical condition and immediate intervention is required as well as hemodynamic stability using fluid and vasopressor. Direct relationship between changes in ETco2 and changes in the cardiac output. We evaluated the study by comparing the effect of using norepinephrine or dopamine on ETco2 of patients with septic shock.

**Methods:**

A clinical trial study was performed on 138 patients with primary diagnosis of septic shock. 70 patients received norepinephrine and 68 patients received dopamine. Patients' end tidal carbon dioxide (ETco2), mean arterial pressure (MAP), pulse rate (PR), arterial blood gas (ABG) levels in two groups were measured and compared at baseline and after 30 and 120 minutes after inotrope infusion. Variables were compared by means of an unpaired student t-test, an unadjusted chi-square test.

**Results:**

138 patients, 70 treated with norepinephrine infusion and 68 with dopamine infusion were included in the study. ETco2 level significantly increased within 120 minutes of treatment in the norepinephrine group (31.10±9.65) compared to the dopamine group (23.71±9.66) (P=0.001). MAP significantly decreased in the group of norepinephrine 30 minutes after treatment (71.71±20.460) (P=0.014) and pulse rate also significantly decreased in the norepinephrine group compared to the dopamine group in 30 minutes (98.07±10.63 vs 106.43±13.54) and 120 minutes (91.15±6.18 vs 103.51±2057) after treatment (P=0.001).

**Conclusion:**

Tissue perfusion and fluid responsiveness of the shock in the norepinephrine group showed improvement. Using ETco2 as a measure for determining volume assessment in patients undergoing mechanical ventilation by septic shock is applicable.

Septic shock is a severe medical emergency and immediate intervention including early diagnosis and treatment of the infection is required by applying appropriate antibiotic therapy and infection source management as well as hemodynamic stability using fluid and vasopressor (1). Its short-term mortality rate is between 45% and 50% (2). Whenever hypotension lasts, despite aggressive fluid therapy, one vasopressor infusion such as norepinephrine or dopamine is employed to maintain MAP at minimum 65 mmHg (3). There is no statistically significant difference between the outcome of applying norepinephrine or dopamine in patients with septic shock (4). However, some studies state that using dopamine in such patients is detrimental (5) and treating with early norepinephrine will lead to improvement of survival (6).

On the other hand, some studies revealed that the mortality rate of patients with septic shock was lower among those who used dopamine (7). Capnography is a noninvasive method used to measure end-tidal carbon dioxide (ETco2) and emergency physicians use it in various critical conditions (8). Abnormal amount of ETco2 at the time of arrival to emergency ward has negative prognosis (9). ETco2 is initially employed in diagnostic treatment of shock in emergency department (10). A lot of studies have shown direct relationship between changes in ETco2 and changes in the cardiac output (11, 12). As the amount of ETco2 decreases, the amount of lactate and mortality rate increases among the patients with shock (13). Therefore, this study aims at comparing the effect of using norepinephrine or dopamine on ETco2 of patients with infectious shock to find out if there is superiority between these two drugs on ETco2 as a measure of tissue perfusion. 

## Methods

**Study patients:** This study is a randomized double-blind controlled clinical trial conducted from January 2016 till January 2018 in the emergency department of Imam Khomeini Educational and Treatment Hospital, the northern largest center with patient referral, Sari, Iran. It was approved by the Ethics Committee of Mazandaran University of Medical Sciences under the code of IR.MAZUMS.REC.95.1547. The research started after registration in the Iranian Registry of Clinical Trials under the code of IRCT2015101924606N1. Finally, written consent was obtained from all the patients or their next of kin. The inclusion criteria comprised patients aged 18 years or older, underwent mechanical ventilation, and required vasopressor (dopamine or norepinephrine) for shock therapy. All patients who underwent mechanical ventilation used Bennett 840 Ventilator for a 2-hour period with the following features: mode: SIMV, Machine rate: 10-12/min, Tidal volume: 8-10cc/kg, PEEP: 3-5, and FIo2<50%. Those with suspected/confirmed infection source, having at least 2 criteria of systemic inflammatory response syndrome (SIRS) including temperature>38°C or <36°C, heart rate>90 beats/min, respiratory rate>20 breaths/min or Paco2<32 mm Hg, white blood cell count>12,000/mm3, <4,000/mm3, or >10% band cell, as well as being in shock condition were considered as patients with septic shock. On the other hand, patients under 18 years of age, with trauma, chronic lung disease, definite brain death, cardiac arrhythmia, referred from other centers, or with more than 2-hour hospitalization in the emergency department were excluded from the study.

**Protocol: **Patients fulfilling the inclusion criteria were assigned into two groups. The first group received dopamine (D) and the second group received norepinephrine (N). Computer-generated randomization was done, and then odd and even patients were allocated in each group. The drugs were prepared with the similar form and volume in syringes and were packed and labeled by a number in opaque envelopes by the principal investigator. Then, the envelopes were given to pharmaceutic nurse in emergency department. Each patient was assigned a number by the emergency department pharmaceutic nurse based on randomization table and consistent with that number, a syringe was ordered by the resuscitation room nurse who was unaware of the type of the medication. The faculty member who was an emergency medicine specialist and was involved in recording the results did not know about the type of the drug, too. The only person who knew the codes was the principal investigator who was not involved in the study process. 

The dosage of the drugs was prescribed according to the estimated weight of the patients. Group D received dopamine from a 5ml solution with 200 mg/ml concentration, Caspian Tamin Pharmaceutical-Co, Iran, with the initial dosage of 5-20 µg/kg/min. Group N received norepinephrine from a 10 ml solution with 1 mg/ml concentration, Mr Sterop-Co, Belgium, with dosage of 0.5 up to 0.19 µg/kg/min. In case the patient suffered from hypotension despite using the maximum dosage, open-label norepinephrine was administered. To increase cardiac output, inotropic agents were used. If vasopressor demand was fulfilled, first open-label medication and subsequently the study solution were stopped. The process took 2 hours and in case of any unwanted complications, the clinical trial medication was stopped and open-label drug was substituted. 

**End points: **Initial outcome was ETco2 rate, and secondary outcome measures included the level of blood pressure, heart rate, O2 saturation, and blood gas condition.

**End points measurement: **ETco2 was measured and recorded with capnometry numerical, model IRMA2+, Phasien Co. Blood pressure, heart rate, O2 saturation, and blood gas conditions were also measured before clinical trial infusion, 30 minutes, and 120 minutes after initiation of the intervention. Furthermore, acute physiology and chronic health evaluation II (APACHE II) as well as simplified acute physiologic score (SAPS II) were measured for each patient when entering the study. 

**Statistical Analysis: **According to the previous studies (14, 15), there were at least 68 patients in each group with 15% difference, power of 80% and two-sided significance level of 0.05%. The data were analyzed according to the intention-to-treat (ITT) analysis. Differences in the primary outcome were analyzed by applying an unadjusted chi-square test. Results were presented as absolute and relative risks with 95% confidence intervals. Other binary end-points were analyzed with the use of chi-square test, and continuous variables were compared by means of an unpaired student’s t-test or a Wilcoxon rank-sum test, as appropriate, using SPSS Version 15.0 (SPSS Inc., Chicago, IL). All reported p- values are two-sided and have not been adjusted for multiple testing. The study statistician and investigators remained unaware of the patients’ treatment assignments while performing the final analyses.

## Results

**Figure1:**
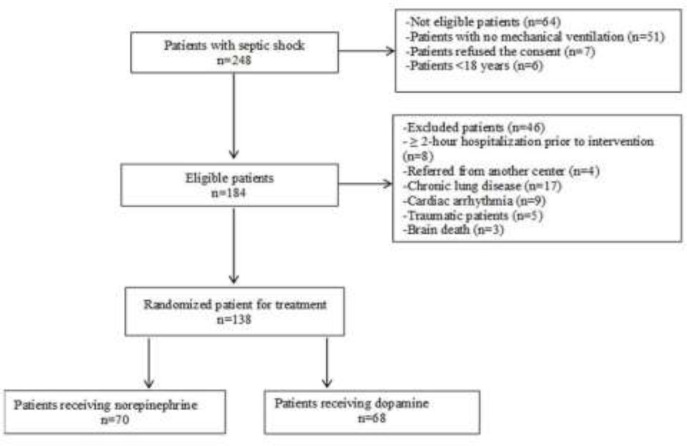
Patient flowchart

Every year 150 patients out of approximately 40000 patients, hospitalized at emergency department of Imam Khomeini Hospital, Sari, suffer from septic shock. During a two-year study, 248 patients with septic shock referred to emergency department, out of whom 138 were recruited in this clinical trial study (figure 1).

Baseline characteristics of the patients are shown in table 1. As it is demonstrated, there are no statistically significant differences between the two groups.

ETco2 rate in both groups has been compared and is displayed in table 2. As it is revealed, at the beginning of the study and 30 minutes after the intervention, there are no significant differences between the two groups, though 120 after the intervention, ETco2 rate has significantly increased in the norepinephrine group. Over the course of time, ETco2 rate in the norepinephrine group has remarkably increased since the initiation of the study up to 120 minutes after the intervention and the difference was statistically significant (P=0.0001). ETco2 in the other group has decreased since the beginning of the intervention up to the next 30 minutes and it was statistically significant (P=0.0007), though it was constant as the time passed. Secondary outcomes that were assessed are shown in table 3. All vital signs of the patients and blood gas values in both groups were similar, but 30-minute MAP in the norepinephrine group increased significantly in comparison to the dopamine group. However, 120 minutes after the intervention, MAP values got close to each other and no significant difference was observed. Heart rate of the patients in 30 and 120 minutes after the intervention, in norepinephrine group significantly decreased in comparison to the other group. Furthermore, O2sat and CO2 pressure in 120 minutes after the intervention significantly increased in comparison to epinephrine group.

**Table 1:** Baseline Characteristics of the Patients

**Variable**		**Dopamine ** **(n=68)**	**Norepinephrine** **(n=70)**	**P value**
Age-year	Median(SD)	75.33(15.17)	66.11(16.91)	0.107
Sex-no. (%)	Male	40 (58.82)	42 (60)	0.888
	Female	28 (41.17)	28 (40)	
APACHE II	Median	31	31	0.664
	Inter quartile range	6.75	13	
SAPS II	Median	73	74	0.554
	Inter quartile range	13.75	23	
Arrhythmia	Beginning: Mean (%)	6 (8.82)	6 (8.57)	0.971
	120 min after intervention: Mean (%)	6 (8.82)	6 (8.57)	0.971
Mortality at 30 days; no. (%)		35 (51.47)	45 (64.28)	0.127

SD, Standard Deviation; APACHE II, Acute Physiology and Chronic Health Evaluation II; SAPS II, Simplified Acute Physiology Score.

**Table 2:** ETco2 Measurement

**P value**	**Dopamine (n=68)**	**Norepinephrine (n=70)**	**Time** **Mean±SD**
0.815	25.84 (9.11)	26.20 (9.06)	Beginning of the intervention
0.074	23.10 (10.61)	26.30 (9.95)	30 min after intervention
<0.001	23.71 (9.66)	31.10 (9.65)	120 min after intervention

SD, Standard Deviation

**Table 3:** Secondary Outcomes and Adverse Events

**P value**	**Dopamine** **(n=68)**	**Norepinephrine** **(n=70)**	**Variable** **Mean±SD**	
0.820	55.06±6.059	54.77±8.489	Beginning	MAP
0.014	61.84±12.276	71.71±20.460	30 min after intervention	
0.928	73.74±10.641	73.50±18.588	120 min after intervention	
0.329	106.69±16.54	109.34±15.27	Beginning	PR
0.001	106.43±13.54	98.07±10.63	30 min after intervention	
<0.001	103.51±2057	91.15±6.18	120 min after intervention	
0.655	96.60±4.03	96.94±4.83	Beginning	O2saturation
0.972	99.04±2.14	99.06±2.13	30 min after intervention	
<0.001	99.98±0.01	99.03±1.62	120 min after intervention	
0.694	7.18±0.152	7.17±0.211	Beginning	Blood PH
0.194	7.20±0.141	7.24±0.186	30 min after intervention	
0.431	7.26±0.132	7.28±0.155	120 min after intervention	
0.588	17.26±4.141	17.67±4.779	Beginning	Blood Hco3
0.055	16.58±2.846	17.89±4.869	30 min after intervention	
0.585	18.99±2.370	18.69±4.023	120 min after intervention	
0.382	47.00±22.373	50.60±25.837	Beginning	Blood Co2 pressure
0.009	40.411±12.186	47.477±18.601	30 min after intervention	
0.009	37.45±12.636	44.47±17.56	120 min after intervention	

SD, Standard Deviation; MAP, Mean Arterial Pressure; PR, Pulse Rate

## Discussion

This clinical trial study revealed that ETco2 rate and Co2 pressure of blood in patients with septic shock receiving norepinephrine were significantly higher than those receiving dopamine. Although some vital signs such as MAP, O2 saturation, blood Hco2, blood PH, and mortality rate of people in the two groups did not have significant differences, the heart rate in patients receiving norepinephrine decreased during time in comparison with the dopamine group. De Backer et al. demonstrated that mortality rate of patients with septic shock receiving dopamine or norepinephrine was not statistically significant, though complications such as cardiac arrhythmia increased in the dopamine group (4). However, in this study arrhythmia rate in both groups were not significantly different which may be due to short period of follow-up (up to 120 minutes). Several studies have shown that using dopamine in patients with shock leads to higher mortality rate, cardiac arrhythmia, and digestive complications (16-18). 

A prognostic marker for mortality in hospital is ETco2 of less than 25 (19). In this study, despite the similarity of the ETco2 in both groups at the beginning of the study and after 120 minutes, ETco2 in the norepinephrine group considerably increased, though it had no association with the mortality rate. More than 5.8% increase in ETco2 rate is indicative of fluid responsiveness of the patients; therefore, cardiac output can be improved by 500 cc increase in isotonic fluid (20, 21). In this study, ETco2 increased in norepinephrine group after the beginning of the study to 120 minutes. Therefore, it can be concluded that cardiac output in these patients has improved and that during shock therapy, one can increase the cardiac output using 500 cc of fluid. Although Guirgis FW et al. in their study revealed that there is no relationship between ETco2 and central venous oxygen saturation (SCVo2) as a clinical improvement marker of the patient in goal of early sepsis therapy, a significant relationship was found between lactate and ETco2. However, at the end, lactate had no significant relationship with ETco2 between 6-hour changes (22). Since our study has been done in a 2-hour period, ETco2 changes as a marker in goal of early sepsis therapy was not assessed. In the study conducted by Rui Q, heart rate of the patients with shock using dopamine infusion significantly increased in comparison to those receiving norepinephrine, but no significant difference was observed in the MAP of the patients in the two groups (16). Our study confirmed these findings, as well. Like any other study, this study had some limitations. First of all, since most of the patients were transferred to ICU after emergency department, they have been followed-up only for 2 hours. Moreover, chronic pulmonary patients were excluded from the study, though patients with acute respiratory distress, pulmonary edema, and pulmonary consolidation syndrome were involved in the study. This could affect the ventilation to perfusion proportion as well as ETco2 rate. Thirdly, this emergency department is the central ward for receiving patients in the north of Iran and these patients had to be excluded from the study; therefore, data collection was hard to do. Hence, further studies are required with a longer period of time as well as considering ETco2 changes as markers in volume assessment in goal of early sepsis therapy. 

During septic shock treatment, heart rate of the patients decreased, while ETco2 significantly increased in the norepinephrine group in comparison to the dopamine group. This can be indicative of improvement in tissue perfusion and fluid responsiveness of the shock in the norepinephrine group, though further studies are required to consider ETco2 as a prognostic marker in treatment of patients with septic shock. Using ETco2 as a measure for determining volume assessment in patients undergoing mechanical ventilation by septic shock is applicable, but in patients with spontaneous respiration and other factors for shock, further studies are required. 
